# The Hours Most Fatal to Life

**Published:** 1876-11

**Authors:** 


### THE HOURS MOST FATAL TO LIFE.

We have ascertained the hours of death in 2,880 instances of all ages, and have arrived at interesting conclusions. We may remark that the population from which the *data* are derived is a mixed population in every respect, and that the deaths occurred during a period of several years. If the deaths of 2,880 persons had occurred indifferently at any hour during the twenty-four years, 120 would have occurred at each hour. But this was by no means the case. There were two hours in which the proportion was remarkably below this, two *minama* in fact—namely, from midnight to 1 o'clock, when the deaths were 83 per cent below the average, and from noon till 1 o'clock, when they were 20 3-4 per cent below. From 3 to 6 o'clock, A. M., inclusive, and from 3 to 7 o'clock, P. M., there is a gradual increase, in the former of 23 1-2 per cent above the average; in the latter of 5 1-2 per cent. The *maximum* of death is from 5 till 6 o'clock, A. M., when it is 40 per cent above the average; the next, during the hour before midnight, when it is 25 per cent in excess; a third hour of excess is that from 9 till 10 o'clock, in the morning, being 17 1-2 per cent above. From 10 A. M., to 3 P. M., the deaths are less numerous, being 16 1-2 per cent below the average, the hour before noon being the most fatal. From 3 o'clock, P. M., to 7 P. M., the deaths rise to 5 1-2 per cent above the average, and then fall from that hour to 11, P. M., averaging 6 1-2 per cent below the mean. During the hours from 9 till 11 in the evening there is a *minimum* of 6 1-2 per cent below the average. Thus the least mortality is during the mid-day hours—namely, from 10 to 3 o'clock; the greatest during early morning hours, from 3 to 6 o'clock. About one-third of the total deaths were children under five years of age, and they show the influence of the latter still more strikingly. At all hours, from 10 o'clock in the morning until midnight, the deaths are at or below the mean; the hours from 10 to 11, A. M., from 4 to 5, P. M., and from 9 to 10, P. M., being *minima*, but the hour after midnight being the lowest *maximum;* at all the hours from 2 to 10, A. M., the deaths are above the mean, attaining their *maximum* at from 5 to 6, A. M., when it is 45 1-2 per cent above.—*London Quarterly Review.*

